# Efficacy and Safety of Fingolimod in Daily Practice: Experience of an Academic MS French Center

**DOI:** 10.3389/fneur.2017.00183

**Published:** 2017-05-05

**Authors:** Thomas Roux, Elisabeth Maillart, Jean-Sébastien Vidal, Sophie Tezenas du Montcel, Catherine Lubetzki, Caroline Papeix

**Affiliations:** ^1^AP-HP, Neurology Department, Pitié-Salpétrière Hospital, Paris, France; ^2^AP-HP, Neurology, Broca Hospital, Paris, France; ^3^AP-HP, Biostatistics Department, Pitié-Salpétrière Hospital, Paris, France

**Keywords:** fingolimod, relapsing-remitting multiple sclerosis, efficacy, annual relapse rate, disability, safety

## Abstract

**Introduction:**

Fingolimod (Fg), a sphingosine 1-phosphate receptor modulator, decreases the annual relapse rate (ARR) in relapsing-remitting multiple sclerosis (RRMS). The aim of this study was to assess the efficacy and safety of Fg in daily practice in patients with RRMS, previously treated with natalizumab (Nz) or not, and systematically followed during at least 1 year.

**Methods:**

Data were collected from the patient files. Primary endpoint was the comparison between the ARR the year before Fg onset and after 1 and 2 years of Fg treatment. The secondary endpoints were the difference between Expanded Disability Status Scale (EDSS) at Fg onset and after 1 and 2 years of treatment, and safety.

**Results:**

In the whole sample, we confirmed Fg efficacy on the ARR (0.895 before vs. 0.364 1 year after, *p* < 0.0001). Between our two groups (with or without Nz before Fg), the ARR was higher in the Nz group during the first year but similar during the second year. The EDSS was stable during the first year of Fg but significantly higher after 2 years (3.33 vs. 3.72, *p* = 0.02). Concerning safety, only three patients had to discontinue Fg because of tolerance issues.

**Conclusion:**

Our study showed that Fg is safe in RRMS and can be used either after first-line treatments or after Nz. However we observed a mild disability progression after 2 years.

## Introduction

Fingolimod (Fg), a sphingosine 1-phosphate receptor modulator, reduces the release of lymphocytes from secondary lymphoid organs, thereby their infiltration in the central nervous system (1). Fg decreases the annual relapse rate (ARR) in relapsing-remitting multiple sclerosis (RRMS) vs. placebo and has shown a superior efficacy against interferon beta-1a (2, 3). Its use has been initially restricted, in Europe, to very active RRMS or after failure of immunomodulatory treatment. Despite this, Fg is actually used after natalizumab (Nz). The aim of this study was to assess the efficacy and safety of Fg in daily practice in patients with RRMS, previously treated with Nz or not, and systematically followed during at least 1 year.

## Materials and Methods

### Population

In this retrospective and monocentric study, every patient fulfilling RRMS 2005 McDonald criteria (4) and treated with Fg (GILENYA) during at least 3 months from January 2012 to December 2013, in Pitié-Salpêtrière MS clinic, was included in our analysis.

### Data Collection

Data were collected retrospectively from MS patient files. Personal characteristics of the patients, previous history of MS, previous and ongoing disease modifying drugs (DMDs), pretreatment blood panel including JCV serology, and all data from the follow-up by the treating neurologist were systematically reported. Patients’ follow-up was scheduled according to the French authority recommendations in our MS clinic. At Fg onset, Expanded Disability Status Scale (EDSS) (5) was scored, and a brain MRI was performed. Then, a biannual visit was scheduled to collect any adverse and serious adverse events, relapses and to score EDSS (6) and disability. A blood panel with lymphocytes count and liver function tests was performed every 3 months the first year, then every 6 months for each patient. A brain MRI was performed every year during treatment. Ophthalmological visit with optical coherence tomography was completed before treatment, 3 months after treatment onset, and then every year. After Fg withdrawal, a biannual visit was scheduled to collect relapses, DMD treatment and to score EDSS. A brain MRI was also performed 6 months after Fg withdrawal. The end of the follow-up was October 2014.

A relapse of MS was defined as occurrence, recurrence, or worsening of symptoms of neurological dysfunction lasting over 24 h and usually ending up in a partial or complete remission (6, 7). Fatigue alone and transient fever-related worsening of symptoms were not considered as a relapse. Symptoms occurring within a month were considered as part of the same relapse. Relapses were diagnosed by the treating neurologist during the follow-up and retrospectively collected according to patients’ files.

The MS course was categorized according to 1996 classification (7). Patient disability was assessed using the EDSS that ranges from 0 (no disability) to 10 (death of the patient), with 0.5-point increment (5). Disability progression was defined as increase of at least 1.0-point EDSS score if EDSS ≤5.5 or 0.5-point if EDSS >5.5 compared to the EDSS value at Fg onset. EDSS was assessed by the treating neurologist during the follow-up and retrospectively collected according to patients’ files.

Due to the amount of missing data (more than 50%), brain MRI results were not analyzed. Safety data were also reported: cardiac events at Fg onset, infections, high blood pressure, macular edema, lymphopenia (<500/mm^3^), elevated liver enzymes (>3 N).

### Study Objectives

The primary objective was to assess the efficacy and the safety of Fg in patients with MS in daily practice. We focused on (i) the comparison between ARR the year before Fg onset and after 1 and 2 years of Fg treatment, (ii) the difference between EDSS at Fg onset and after 1 and 2 years of treatment, (iii) the reasons to Fg discontinuation, and (iv) the number and the types of adverse events.

### Statistical Analysis

Data are expressed as the mean (SD) [M (SD)] or percentages and numbers [*n* (%)]. The sample was divided in two subgroups according to the use of Nz during the 6 months preceding Fg onset (Nz and no-Nz groups). General characteristics were calculated in the whole sample and according to Nz groups, and comparison was made using χ^2^ test or *T*-test for categorical and continuous variables, respectively.

The ARR was calculated in the whole sample and according to the Nz groups during the year before Fg, during the first year after Fg onset, and during the second year after Fg onset, and comparisons between Nz groups were made with mixed model with ARR as dependent variable and random intercept.

The characteristics of the sample were then analyzed according to the disability progression, defined as an increase of EDSS 1-point during the first year of Fg treatment. Comparisons were made with non-parametric statistics (Wilcoxon signed-rank test and Fisher exact test).

Statistical analysis was performed with R (8). *p*-Values <0.05 were considered significant.

## Results

### Analytical Sample

**Figure 1:**
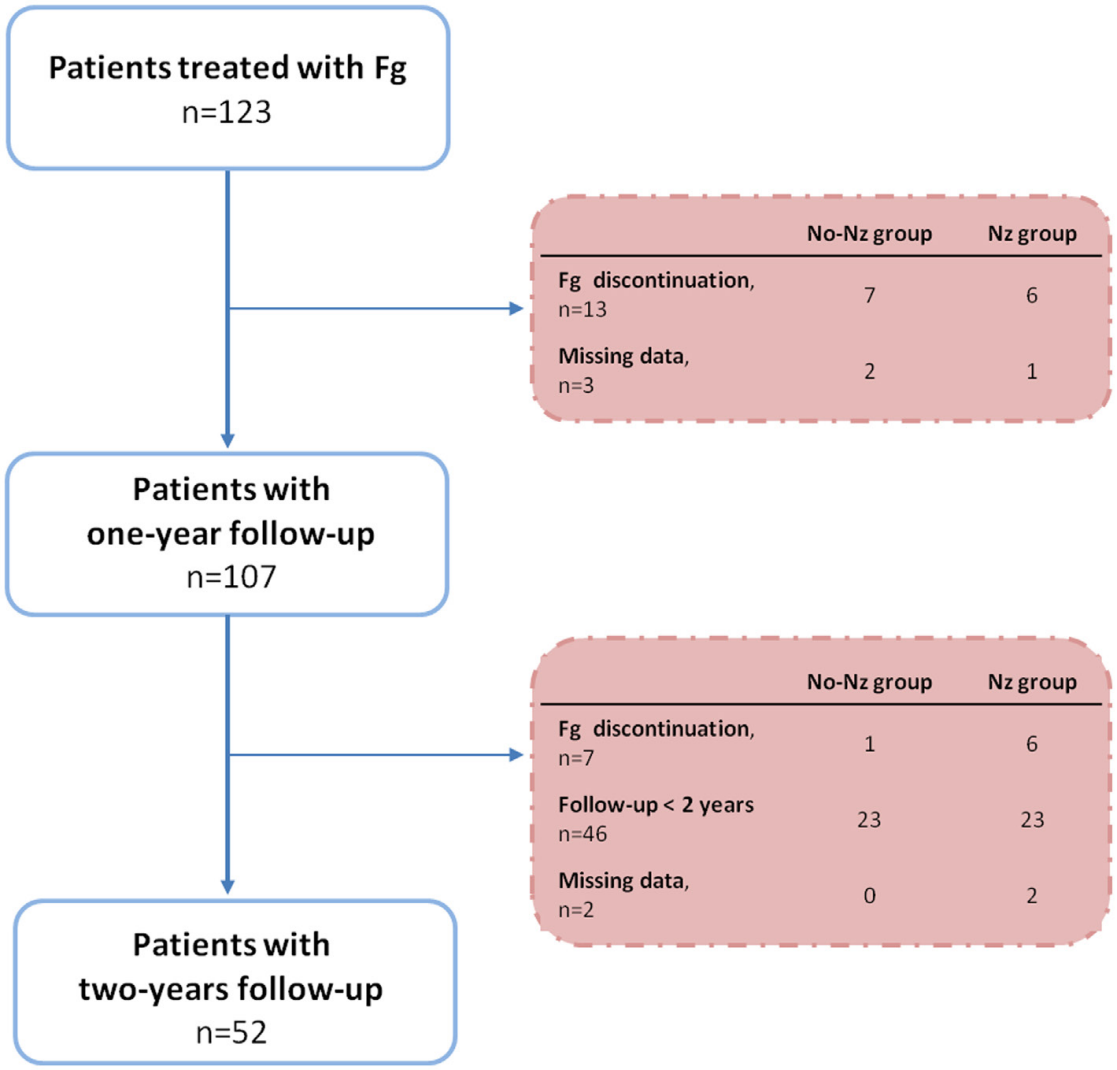
**Flowchart**. Fg, fingolimod; Nz, natalizumab.

Among the 123 patients treated with Fg, the ARR after Fg onset was missing for 16 of them. Thus, the ARR was analyzed in 107 patients, 71 women and 36 men (Figure 1). Compared to the 107 analyzed patients, the 16 non-analyzed patients were older at MS diagnosis [mean = 30.8 (SD = 8.0) vs. 25.6 (8.3), *p* = 0.01] and started Fg older [42.8 (10.3) vs. 35.1 (8.5), *p* = 0.004] (Table 1).

**Table 1:** **Comparison of analyzed and non-analyzed patients**.

Characteristics, M (SD)	Analyzed	Non-analyzed	p*
	*N* = 107	*N* = 16	
Women, % (*N*)	66.4 (71)	56.2 (9)	0.57
Age at MS onset	25.6 (8.3)	30.8 (8.0)	0.01
Disease duration	9.43 (4.92)	12.1 (6.4)	0.16
Age at Fg onset	35.1 (8.5)	42.8 (10.3)	0.004
Duration of Fg treatment (months)	16.2 (8.6)	7.41 (1.18)	0.14

*Wilcoxon signed-rank test of Fisher’s exact test.

*Fg, fingolimod*.

### Population

The mean age at Fg onset was 35.1 (8.5) years, the mean disease duration was 9.43 (4.92) years, and JCV serology was found positive in 71 (92.2%) patients tested (*n* = 77). The mean follow-up was 22.0 (7.0) months.

Fingolimod was introduced (i) after injectable DMD (*n* = 42), (ii) after immunosuppressants (*n* = 54) [azathioprine (*n* = 1), cyclophosphamide (*n* = 1), Nz (*n* = 52)], or (iii) in naïve patients (*n* = 11). The demographic and clinical characteristics according to Nz treatment before Fg are summarized in Table 2. Compared to the patients not previously treated with Nz, the patients previously treated with Nz had a longer disease duration [11.5 (4.8) vs. 7.48 (4.23), *p* ≤ 0.0001] and a higher EDSS [3.93 (2.09) vs. 3.26 (1.94), *p* < 0.09] at Fg onset. In the Nz group, the mean washout duration was 101 (30) days. The switching protocol was carefully applied in our unit and almost every patient started Fg 3 months after the last Nz infusion. Patients discontinued Nz because of positive JCV serology (*n* = 46), drug inefficacy (*n* = 1), based on a personal preference (*n* = 3), adverse effects (*n* = 1), unknown reason (*n* = 1).

**Table 2:** **Baseline characteristics in the whole sample and according to Nz groups**.

Characteristics, M (SD)	Whole sample	No-Nz group	Nz group	p*
	*N* = 107	*N* = 55	*N* = 52	
Women, % (*N*)	66.4 (71)	70.9 (39)	61.5 (32)	
Age at MS onset	25.6 (8.3)	27.7 (8.7)	23.3 (7.3)	0.006
Disease duration	9.43 (4.92)	7.48 (4.23)	11.5 (4.8)	<0.0001
Age at Fg onset	35.1 (8.5)	35.4 (8.4)	34.8 (8.6)	0.69
EDSS at Fg onset	3.58 (2.03)	3.26 (1.94)	3.93 (2.09)	0.09

*χ^2^ test or T-test.

*M (SD), mean (SD); % (*N*), percentage (number); Nz, natalizumab; Fg, fingolimod; EDSS, Expanded Disability Status Scale*.

### Clinical Efficacy: ARR and EDSS

Concerning the whole population, 42 patients (39.3%) had one or more relapse under Fg including 24 patients (22.4%) with at least one relapse within the 3 months after Fg onset. In the Nz group, nine patients (14.8%) relapsed during the washout between Nz and Fg, but their ARR remains similar to other patients at 1 and 2 years (data not shown). Eleven patients (21.2%) experienced at least one relapse within the 6 months after Fg start, and four patients (7.7%) experienced their first relapse between 6 and 12 months after Fg start.

In the whole sample, the ARR was 0.895 (SE = 0.898) during the year before Fg onset, 0.364 (0.620) during the first year after Fg onset (*p* < 0.0001) and 0.423 (0.696) during the second year after Fg onset (Table 3).

**Table 3:** **ARR according to Nz groups**.

Population	Year − 1 before Fg	Year + 1 after Fg	Year + 2 after Fg

	*n* = 107	*n* = 107	*n* = 52
Whole sample	0.90 (0.90)*	0.36 (0.62)	0.42 (0.70)
Nz group	0.34 (0.63)	0.42 (0.67)	0.57 (0.84)
No-Nz group	1.40 (0.81)*	0.31 (0.57)	0.31 (0.54)
*p*^†^	<0.0001	<0.0001	0.52

Comparison to Year + 1 after Fg, *p < 0.0001.

*^†^Comparison between the Nz and no-Nz groups using mixed model*.

*ARR, annual relapse rate; Nz, natalizumab; Fg, fingolimod*.

In the Nz group, the ARR was 0.340 (0.626) the year before Fg onset. Compared to the year before Fg onset, there were no significant changes during the first year of Fg [0.423 (0.667), *p* = 0.54]. Compared to the first year of Fg, there were no significant changes during the second year of Fg [0.565 (0.843), *p* = 0.41].

In the no-Nz group, the ARR was 1.400 (0.807) during the year before Fg onset. Compared to the year before Fg onset, the ARR was significantly lower during the first year of Fg [0.309 (0.573), *p* < 0.0001]. Then, the ARR remained stable during the second year of Fg [0.310 (0.541), *p* = 0.96].

Compared to the patients who did not receive Nz before Fg, those who received Nz before Fg had, on average, a lower ARR during the year before Fg onset [0.340 (0.626) vs. 1.400 (0.807), *p* < 0.0001] and a higher ARR during the first year of Fg [0.423 (0.667) vs. 0.309 (0.573), *p* < 0.0001]. However, there was no significant difference between the two groups for the ARR during the second year of Fg [0.565 (0.843) vs. 0.310 (0.541), *p* = 0.52].

In the whole sample, the EDSS was 3.33 (1.94) the year before Fg onset. Compared to the year before Fg onset, the EDSS was not different after 1 year of Fg [3.58 (2.03), *p* = 0.57] but significantly higher after 2 years of Fg [3.74 (2.00), *p* = 0.02].

Univariate analysis was performed to identify factors associated with the increase of the disability (Table 4). Sex, age, disease duration, ARR the year before Fg, and EDSS at Fg onset were all not significant.

**Table 4:** **Sample characteristics according to disability progression (based on EDSS change at 1 year after Fg onset)**.

Variables and values, M (SD)	No disability progression	Disability progression	p*
	*N* = 86	*N* = 18	
Age at MS onset	25.8 (7.7)	26.1 (10.9)	0.71
Women, % (*N*)	67.4 (58)	61.1 (11)	0.60
Disease duration before Fg	8.92 (4.71)	11.3 (5.2)	0.07
Age at Fg onset	34.8 (8.4)	37.4 (8.5)	0.24
EDSS before Fg	3.60 (2.00)	3.47 (2.29)	0.63
ARR before Fg	0.97 (0.92)	0.67 (0.77)	0.22

*Wilcoxon signed-rank test or Fisher’s exact test.

*ARR, annual relapse rate; Nz, natalizumab; Fg, fingolimod; EDSS, Expanded Disability Status Scale*.

### Treatment Discontinuation

Twenty patients (18.6%) discontinued Fg during the period of observation, because of adverse events (*n* = 3), drug inefficacy (*n* = 6), based on personal decision (*n* = 6), or because of an evolution toward a progressive form of MS (*n* = 5).

There was no statistical difference between the EDSS during Fg and the EDSS 6 months after Fg discontinuation [5.2 (2.3) vs. 5.6 (2.2), *p* = 0.63]. From seven patients with relapses during treatment with Fg, four patients experienced relapses again after Fg withdrawal. Two patients, who have not relapsed during treatment with Fg, remained relapse free after Fg cessation.

### Safety

We observed 13 episodes of transient asymptomatic bradycardia without atrioventricular block at Fg introduction, 5 lymphopenia (<500/mm^3^), 3 hepatic cytolysis (>3 N), 10 infections including 5 with hospitalization, 1 macular edema, 1 transient dizziness, and 1 suicide attempt. Three patients had to discontinue Fg because of tolerance issues: one severe lymphopenia (<200/mm^3^), one macular edema, and one transient dizziness.

## Discussion

This retrospective study including all MS patients treated by Fg in Salpêtrière Hospital provides real-life information about Fg use, especially after Nz switch. In this cohort of 107 patients treated with Fg, the ARR was reduced the first year of Fg, and stable thereafter. Actually, two different groups were identified: the Nz group, with the patients previously treated with Nz, and the no-Nz group, with the patients who did not received Nz. In the Nz group, stability of the ARR between the year before Fg onset and the next 2 years can be explained by the low ARR observed with Nz treatment (ARR = 0.34 ± 0.63). On the contrary, in the no-Nz group, the ARR during the year before Fg onset was 1.4 ± 0.8. A reduction of the ARR after 1 year was observed. This efficacy was similar to phase III study (2). Interestingly, the ARR during Fg treatment was similar between our two groups in the second year but not in the first year where it was higher in the Nz group. Not all the patients achieved 2 years of Fg at the time of the analysis (Figure 1). Thus, our sample size is smaller the second year. Moreover, the reduction of the ARR during the second year of Fg might be partly explained by the drop out of the poor responders (6 among 123 patients). Still, within the subgroup (Nz group), Fg seemed to be as efficient as was Nz.

Two recent studies, comparing the efficacy of Fg and Nz retrospectively, have conflicting results. The first one was in favor of a better efficacy of Nz on the ARR and the MRI activity (9). The second one, using the Danish MS registry and comparing 464 patients under Nz and 464 under Fg reported similar efficacy on relapses for both treatments (10). As for our study, it showed that Fg used after Nz treatment can maintain the efficacy of the previous treatment. Moreover, the study using the Italian MS database published in 2015 showed the same trend (11). Among the 433 patients analyzed in this study, 135 switched from Nz to Fg and 298 to interferon/glatiramer acetate. There was a significant lower incidence of relapses in the patients treated with Fg in comparison with those treated with interferon beta/glatiramer acetate after the switch. Finally, it seems that Fg is a reasonable treatment either after first-line DMD or after more aggressive treatment like Nz.

The EDSS was stable after 1 year but slightly increased after 2 years in the whole cohort, which was not found in FREEDOMS trial (EDSS stable at 2.3 after 2 years) (3). One explanation could be that patients in our study were more severe, with a higher previous EDSS (3.33 ± 2 vs. 2.3) and a longer disease duration (9.4 ± 5 vs. 8 ± 6 years). Indeed, it would mean that Fg could be more efficient if it is started in the beginning of the disease. But we should stress that the follow-up in our study was of 2 years, which is quite superior to other Fg cohorts in literature (2, 11–23), except two (3, 9). Still, we could not find any factor associated with EDSS worsening.

Brain MRI results were not analyzed in this study due to the amount of missing data [78 missing MRIs after 1 year of Fg (63%)]. It stresses the fact that brain MRI is not performed as scheduled despite its high priority. Yet, brain MRI is a key point in the evaluation of a treatment efficacy, like it is used in the modified Rio score with interferon treatment (24). Indeed, in a real world clinical setting prospective observational multicenter study including 142 RRMS patients treated with Fg, Totaro et al. showed a reduction of the ARR after Fg start (25). But the brain MRI monitoring revealed that new contrast enhancing lesions were more common in patients undergoing previous Nz treatment compared to others (26.7 vs. 15.3%, *p* = 0.046). Moreover, brain MRI is essential in the detection of progressive multifocal leukoencephalopathy, especially in those patients who had Nz before.

The JCV serology was found positive in 92.2% of the patients who were tested, which is more than expected in the general and MS populations (26). This result could be explained by the fact that JCV serology became available in France in September 2011. This test has changed our practice: from that date, the patients with a positive JCV serology were often switched for Fg.

In the Nz group, the mean washout duration was 101 (30) days, which is similar to others’ studies in literature (12–15, 18–20, 23, 27), but the duration of the follow-up was superior (21 ± 7 months) (12–16, 18, 19, 22, 23). Relapse during switch occurred in 15% of patients, but the ARR remains identical at 1 and 2 years (data not shown). In the Italian study, the median of washout period was 5.1 (q1–q3 = 3.5–10.5) months for the patients who switched from Nz to Fg, and 19.4% of these patients experienced a relapse (11). Altogether, these data confirmed that a short period of washout is beneficial.

Besides, the first relapse of the patients previously treated with Nz mostly happened within the 6 months after Fg start. This is consistent with the results of a large French post-marketing cohort including 715 patients who stopped Nz (28). In this study, most of relapses occurred between 3 and 5 months after Nz discontinuation regardless of the treatment afterward.

As expected, the safety is correct, with only three Fg discontinuation after adverse events, as described in other studies (2, 3, 14, 16, 18, 27, 29). This is very reassuring considering the 2-year follow-up of our study.

We are aware that our study has many biases, including a retrospective analysis and a small size of the cohort. However, it provides real-life information about Fg treatment, especially with the patients who were not included in the phase III studies (i.e., switch from Nz). It highlights that, in daily practice, Fg is efficient and safe in RRMS. Our study also revealed that Fg can be used either after first-line DMDs or after Nz. However, we observed a mild disability progression after 2 years. Controlled randomized trials, with longer follow-up and larger samples, are needed.

## Ethics Statement

Clinical data were obtained in accordance with ethical standards laid down in the 1964 Declaration of Helsinki and its later amendments. All patients were informed and approved to participate to this study prior inclusion. In this retrospective clinical study, no authorization from an ethical committee was required.

## Author Contributions

TR, EM, and CP acquired and analyzed the data. J-SV, STM, and CL analyzed the data. All the authors revised and approved the manuscript.

## Conflict of Interest Statement

EM has received conference fees for Biogen, Genzyme, Novartis, and Teva. CP served on the scientific advisory board for Bayer Schering, Novartis, and Teva and was employed for educational activities from Bayer Schering, Sanofi-Genzyme, Biogen Idec, Novartis, Merck Serono, and Roche. CL served on the scientific advisory board for Vertex, Biogen, Novartis, and Genzyme; served on the editorial board for MSJ and MSJ and Related Disorders; was associate editor for Brain; consulted for Biogen, Roche, and Genzyme; and had a scientific collaboration with EMD Serono. The other authors declare no conflict of interest.

## Abbreviations

ARR, annual relapse rate; DMD, disease-modifying drug; EDSS, Expanded Disability Status Scale; Fg, fingolimod; Nz, natalizumab; RRMS, relapsing-remitting multiple sclerosis.
